# Balancing clinical benefits and behavioral risks of obesity-focused management in youth-onset type 2 diabetes: A narrative review

**DOI:** 10.17925/EE.2026.22.2.6

**Published:** 2026-10-14

**Authors:** Rutha Chivate, Caroline Christian, Abigail Hartman, Afsana Dhali, Andrea B Goldschmidt, Mary Ellen Vajravelu

**Affiliations:** 1. University of Pittsburgh School of Medicine, Pittsburgh, PA, USA; 2. Department of Psychiatry, University of Pittsburgh School of Medicine, Pittsburgh, PA, USA; 3. Diabetes Wraparound Program, UPMC Children’s Hospital of Pittsburgh, Pittsburgh, PA, USA; 4. University of Pittsburgh, Pittsburgh, PA, USA; 5. Department of Pediatrics, Division of Pediatric Endocrinology and Center for Pediatric Obesity and Metabolism, University of Pittsburgh School of Medicine, Pittsburgh, PA, USA

**Keywords:** Diabetes mellitus, type 2, diagnostic screening programs, feeding and eating disorders, glucagon-like peptide 1 receptor agonists (GLP-1 RA), obesity, pediatrics

## Abstract

Weight management is a key treatment goal for youth-onset type 2 diabetes, a condition that is strongly linked with obesity. For this reason, glucagon-like peptide-1 (GLP-1) receptor agonists, with their glycemic- and weight-reducing effects, have been increasingly used in adolescents and young adults with type 2 diabetes. However, because disordered eating behaviors and eating disorders are also relatively common in this population, use of appetite-suppressing medications such as GLP-1 may have unintended psychological or behavioral consequences. In this narrative review, we summarize the epidemiology of disordered eating behaviors and eating disorders among youth with type 2 diabetes and the limited evidence related to disordered eating and GLP-1 use. We end with suggested strategies for clinicians to identify and care for youth with disordered eating behaviors or eating disorders in the context of type 2 diabetes. Future research should prioritize the creation and validation of a disordered eating screener that is appropriate for youth with type 2 diabetes undergoing medical weight management, as well as identify factors associated with positive versus negative eating behavior responses to GLP-1.

Youth-onset type 2 diabetes is increasingly common in the USA, with incidence exceeding that of type 1 diabetes among 15–19-year-old adolescents and prevalence projected to increase by 673% by 2060.^1,2^ This growth is particularly worrisome due to the severity of youth-onset type 2 diabetes, which is more aggressive than adult-onset type 2 diabetes and carries a high risk of comorbid hypertension, dyslipidemia and diabetic kidney disease in young adulthood.^3^ One major risk factor for type 2 diabetes is pediatric obesity, defined as body mass index (BMI) ≥95th percentile for age and sex. At the time of type 2 diabetes diagnosis, approximately 80% of youth have obesity, which carries an increased risk of cardiovascular and kidney disease above and beyond a diagnosis of type 2 diabetes alone.^4–6^ Obesity management is, therefore, a key goal of type 2 diabetes care, including both healthy lifestyle changes and careful medication selection to optimize glycemic and weight outcomes.^7^ Glucagon-like peptide-1 (GLP-1) receptor agonists, which were originally developed to support glycemic control, also have robust beneficial effects on weight, unlike metformin and insulin. Indeed, given their positive glycemic- and weight-lowering effects, there have been calls to alter guidelines to prioritize early combination therapy including GLP-1 as first-line therapy for youth with type 2 diabetes.^8^

Currently, four different GLP-1 medications have been approved by the U.S. Food and Drug Administration for use in youth with type 2 diabetes (liraglutide, exenatide, dulaglutide, and tirzepatide – a dual GLP-1 and glucose-dependent insulinotropic polypeptide [GIP] agonist).^9–12^ Semaglutide is approved for weight management in youth with obesity, and a recent clinical trial demonstrated glycemic efficacy in youth with type 2 diabetes as well.^13^ While average BMI reduction varies by formulation, tirzepatide results in the greatest reduction (15.1% BMI reduction at 1 year) relative to other formulations approved for type 2 diabetes, although head-to-head pediatric trials are lacking.^12^ Uptake of GLP-1 has been increasing, with more than one-third of youth with type 2 diabetes having a GLP-1 dispensed to them in 2023, among both Medicaid- and commercially insured youth.^14^

Despite the enthusiasm for glycemic- and weight-related efficacy of GLP-1, very little is known about their impact on disordered eating behaviors or risk of eating disorders.^15^ Eating disorders are serious, neurobiologically based psychiatric illnesses characterized by disordered eating behaviors, distorted cognitions around food, eating and body shape/weight, and oftentimes, difficulties with weight regulation.^16^ Even in the absence of meeting full diagnostic criteria for an eating disorder, subthreshold disordered eating behaviors cause significant distress and impairment and can be associated with the same medical risks and consequences as full-syndrome disease states.^17,18^ In this narrative review, we summarize the epidemiology, outcomes, and assessment of disordered eating behaviors and eating disorders in the context of youth-onset type 2 diabetes, with specific attention to evidence related to GLP-1 use whenever possible based on the current literature. We end with a summary of evidence gaps and future directions for research and clinical care.

## Methods

A literature search of PubMed and PsycInfo databases was completed in December 2025 with assistance from a research librarian and updated in April 2026. For PubMed, the following search query was used: (((‘Diabetes Mellitus, Type 2’[Mesh]) OR ‘type two [sic] diabetes’ OR ‘Obesity’[Mesh]) AND ((‘Pediatrics’[Mesh]) OR ‘Adolescent’[Mesh] OR ‘teenager’)) AND ((((‘Metformin’[Mesh]) OR ‘Phentermine’[Mesh]) OR ‘Sodium-Glucose Transporter 2 Inhibitors’[Mesh]) OR ‘Glucagon-Like Peptide-1 Receptor Agonists’[Mesh]) OR ‘Insulin’[Mesh] AND ((‘Disordered Eating Behavior’[Mesh]) OR ‘Feeding and Eating Disorders’[Mesh] OR ‘eating disorder’). For PsycInfo, the search query included: ‘disordered eating’ or ‘Eating Disorders’ and ‘Pediatrics’ or ‘adolescent’ and ‘Type 2 Diabetes’ or ‘Obesity’ or ‘Childhood Obesity.’ This generated 495 references from PsycInfo and 418 references from PubMed. Initial review of the reference title and abstract was divided across all authors. References were considered for inclusion if they were written in English and addressed disordered eating behaviors or eating disorders (‘disordered eating,’ ‘binge eating’ or ‘eating disorder’) among youth (ages 12–18) with type 2 diabetes. If there was uncertainty about eligibility, the full text was reviewed, and inclusion or exclusion was determined by consensus among all authors. Of the 11 manuscripts identified for full-text review, 7 were included; excluded manuscripts were reviews without a focus on disordered eating or eating disorders (n=3) and a study unrelated to youth-onset type 2 diabetes (n=1).^19–25^ One additional manuscript identified via the systematic review of disordered eating behaviors and eating disorders in youth with type 2 diabetes by Mateo et al. was included.^25,26^

## Prevalence and consequences of eating disorders and disordered eating behavior in individuals with type 2 diabetes

Among youth and young adults with type 2 diabetes, the prevalence of disordered eating behaviors has been estimated to be as high as 50%, compared with a prevalence of 21% among youth and young adults with type 1 diabetes.^19^ In the population-based prospective SEARCH for Diabetes in Youth cohort, youth with type 2 diabetes with disordered eating behaviors (versus without) were found to have higher BMI, lower quality of life and more depressive symptoms, as well as more severe experiences of diabetes stigma.^19,20^ The SEARCH for Diabetes in Youth study also demonstrated that disordered eating behaviors were associated with food insecurity among youth with type 2 diabetes, with a gradient of higher scores on the Diabetes Eating Problem Survey-Revised (DEPS-R) in the setting of more severe food insecurity.^20,21^ While full-syndrome eating disorders have been less thoroughly studied in the context of pediatric type 2 diabetes, the prevalence of binge-eating disorder has been estimated to be as high as 25% in adults with type 2 diabetes.^27^ A higher risk of type 2 diabetes among individuals with eating disorders has also been described, with adults with binge-eating disorder or bulimia nervosa having a higher lifetime prevalence of type 2 diabetes versus adult peers without eating disorders (5.2% versus 1.7%), suggesting a bidirectional relationship.^28^

For adolescents with type 2 diabetes, disordered eating behaviors are associated with poor glycemic control, increasing the risk of diabetes-related complications. One factor contributing to worsened glycemic control is insulin restriction, which is more prevalent in individuals with type 1 diabetes but has also been observed in those with type 2 diabetes.^19^ Furthermore, dysregulated glucose metabolism can contribute to the onset of binge-type eating behaviors as seen in binge-eating disorder and bulimia nervosa, which can then worsen complications in a positive feedback loop.^28^ Individuals with disordered eating behaviors are also more likely to experience depressive symptoms, adding to the already high risk of depression for adolescents with type 2 diabetes.^19,29–31^

## Prevalence and consequences of eating disorders and disordered eating behavior in higher-weight youth

Obesity is pervasive among youth with type 2 diabetes, and disordered eating behaviors with or without a formal eating disorder diagnosis are more common among higher-weight youth than their lower-weight peers.^4,5,32^ This likely emerges from shared risk factors for obesity and disordered eating, including dieting, experiences of weight-related stigma and body dissatisfaction.^33^ Binge and loss of control eating, characterized by the subjective experience of being unable to control what or how much one is eating with or without ingestion of an objectively large amount of food, respectively, are particularly prevalent among youth with overweight and obesity, occurring in 26.3% across 36 studies included in a meta-analysis.^34^

**Figure 1:**
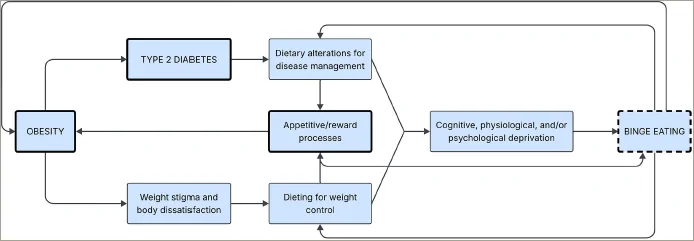
Conceptual model depicting biological, behavioral and psychosocial processes by which obesity, binge eating and type 2 diabetes are related

*Boxes with thicker lines indicate outcomes impacted by glucagon-like peptide-1 (GLP-1) receptor agonists, with thicker solid lines representing known impacts and thicker dashed lines representing putative impacts requiring further study*

The high prevalence of eating disorder symptoms among individuals with higher weight (with or without type 2 diabetes) may be attributed to a variety of risk factors specific to this population, including family history, environmental factors, weight stigma, weight-related teasing, internalized weight bias, increased dieting attempts and weight cycling.^35–38^ Engaging in restrictive dieting behaviors is one of the strongest risk factors for eating disorders among higher-weight youth and is highly relevant among youth with type 2 diabetes and obesity, whose treatment goals may include dietary restriction for both weight loss and glycemic control.^36^ For example, recommendations to manage diet, such as reducing intake of high-carbohydrate foods, may increase feelings of deprivation among vulnerable youth, thereby contributing to further binge eating (*Figure 1*). However, while weight control efforts by adolescents have been linked with higher eating disorder risk, medically supervised weight management interventions may in fact be protective.^39^ A scoping review of 126 behavioral intervention trials including children and adolescents with overweight and obesity found that psychosocial outcomes generally improved or remained unchanged in the intervention arm as compared with controls, although the data were limited by significant heterogeneity in assessments.^39^

## Evaluating and addressing risk for disordered eating behaviors and eating disorders among youth with type 2 diabetes

There are a few validated screening tools for detecting eating disorder risk in youth with normal or high BMI, with screeners often administered to youth who are already showing major physical signs or symptoms of eating disorders, such as rapid weight loss or gain, pubertal delay, pronounced deviation from the growth curve, bradycardia or irregular menstruation.^38^ Existing screening tools are limited by a focus on a finite set of symptoms (e.g. symptoms of restrictive eating or binge eating, but not both). They are also validated primarily among females with low weight and not in racially or ethnically diverse youth (a gap that is particularly relevant in youth-onset type 2 diabetes, which is least common among non-Hispanic White youth).^40^ Diabetes-specific screeners place an emphasis on the use of insulin, which is not universal among youth with type 2 diabetes. Currently validated assessment tools are lengthy, complex to score, do not generate diagnoses and require training to administer, so they are not feasible for use as screening tools.

*Table 1* provides a summary of disordered eating assessment methods used in prior studies, including youth with type 2 diabetes, as well as examples of screening tools used for youth with type 1 diabetes or obesity. Disordered eating assessment methods for youth with type 2 diabetes in prior studies included: DEPS-R, which was validated in adolescents with type 1 diabetes and is more appropriate for youth using insulin; questions from the Youth Risk Behavior Surveillance System (YRBSS), not a formal screening tool, with a focus on weight management worries, goals and attempts; the Youth-Eating Disorder Examination Questionnaire (Y-EDEQ), which evaluates restraint and eating, weight and shape concerns; and the Eating Disorder Diagnostic Scale (EDDS), which assesses attitudinal symptoms of anorexia nervosa and bulimia nervosa, as well as episodes of uncontrollable consumption of large amounts of food and compensatory behaviors.^26,41–44^ For comparison, one survey validated for use in youth with type 1 diabetes (mSCOFF [modified SCOFF]) and two for use in adolescents with obesity but without diabetes (Adolescent Binge Eating Scale [ADO-BED] and Children’s Brief Binge-Eating Questionnaire [CBBEQ]) are also included in *Table 1*.^19,22–24,26,41–48^ As shown in the table, modifications and additional studies would be required to ensure these screening tools are appropriate for youth with type 2 diabetes, who may use a variety of medication regimens that affect appetite and/or weight, including metformin, insulin and GLP-1.

In addition to the appropriateness of a given tool, another important consideration when screening for eating disorders is the availability of specialized care to which clinicians can refer as needed. Most clinicians who have not sought specialized training in eating disorder treatment have limited comfort and ability to recognize and manage eating disorder symptoms.^49,50^ Furthermore, the lack of referral options and expert consultants in many communities, fear of introducing iatrogenic effects or stigmatizing patients and limited self-efficacy lead many providers to avoid asking questions about eating behaviors or weight at all.^51^ Thus, while validated and easily disseminated screening tools are needed for this population, this should be paired with training for clinicians and facilitated referral processes.

**Table 1:** Limitations of existing questionnaires previously used to assess disordered eating among youth with type 2 diabetes, type 1 diabetes and obesity^19,22–24,26,41–48^

Questionnaire	Details	Evidence in pediatric type 2 diabetes	Limitations for use in type 2 diabetes
DEPS-R^41^	16-item survey Validated in adolescents with type 1 diabetes Assesses the frequency of behaviors in the past month Includes items related to ketones and insulin use	50.3% of TODAY study participants (adolescents with recently diagnosed type 2 diabetes) had elevated scores ≥20^19^	Limited relevance to youth not using insulin and at lower risk for ketosis or ketoacidosis Losing weight may be an appropriate goal in the setting of obesity
YRBSS,^42^ adapted^26^	Subset of a larger survey of behaviors, not a true screening tool Descriptive, not validated for detection of eating disorders Assesses self-perception and worry about weight, weight management goals and weight loss attempts	Females with type 2 diabetes are more likely than females with type 1 diabetes to engage in maladaptive eating behaviors, including fasting or using diet aids such as pills without a doctor’s advice^26^	Does not include items about the use of doctor-prescribed medication for weight management No cutoff for detection and referral for evaluation of eating disorders
Y-EDEQ^43^	39-item survey Assesses restraint, eating concern, weight concern and shape concern Subclinical binge eating defined as reporting 1–3 episodes in the past month and clinical binge eating defined as ≥4 episodes	At baseline, 6% of youth enrolled in the TODAY study had clinical binge eating and 20% had subclinical levels of binge eating, associated with extreme obesity, depressive symptoms and impaired quality of life^23^ Subclinical binge eating in parents was associated with subclinical binge eating in adolescents in the TODAY study at baseline^22^	Some items (e.g. ‘definite rules regarding your eating’ and ‘Has thinking about food... made it very difficult to concentrate...’) may be difficult to interpret in the setting of dietary recommendations for diabetes Low accuracy for detecting loss of control eating in younger children with overweight/obesity
EDDS^44^	23-item survey Assesses attitudinal symptoms of anorexia nervosa and bulimia nervosa over the past 3-month period, frequency of uncontrollable consumption of a large amount of food and frequency of compensatory behaviors	Binge eating present in 10% of youth in the TODAY study at baseline and stable over longitudinal follow-up; diagnosable eating disorder (e.g. binge-eating disorder) present in 1.4% at baseline^24^	Insulin underdosing or omission not specified in EDDS
mSCOFF^45,46^	5-item survey, with the fifth item modified from the original to assess insulin omission Validated among adolescents with type 1 diabetes	None	Not relevant for youth not using insulin Potential appropriateness of recent weight loss among higher-weight youth not considered
ADO-BED^47^	6-item survey (with subitems) to evaluate binge eating in adolescents Initially evaluated in adolescents seeking obesity management	None	Nothing specific to living with diabetes Eating while not hungry may be necessary in the setting of hypoglycemia
CBBEQ^48^	7-item survey to evaluate hunger, binge eating and loss of control eating Initially evaluated in adolescents seeking obesity management	None	Nothing specific to living with diabetes Eating while not hungry may be necessary in the setting of hypoglycemia

*TODAY: Treatment Options for type 2 Diabetes in Adolescents and Youth; ClinicalTrials.gov identifier: NCT00081328*

*ADO-BED = Adolescent Binge Eating Scale; CBBEQ = Children’s Brief Binge-Eating Questionnaire; DEPS-R = Diabetes Eating Problem Survey-Revised; EDDS = Eating Disorder Diagnostic Scale; mSCOFF = modified SCOFF ED Screening Questionnaire; TODAY = Treatment Options for type 2 Diabetes in Adolescents and Youth; Y-EDEQ = Youth-Eating Disorder Examination Questionnaire; YRBSS = Youth Risk Behavior Surveillance System.*

## Disordered eating behaviors or eating disorders in the setting of glucagon-like peptide-1 use for type 2 diabetes or obesity management

Due to GLP-1-induced changes in appetitive processes such as food cue reactivity, hunger, satiety and cravings, all of which are putative mechanisms underlying dysregulated eating behaviors, it is possible that some forms of maladaptive eating, such as binge-type eating, may improve with GLP-1 use.^52,53^ While early evidence in adults using GLP-1 suggests improved or unchanged disordered eating behaviors, with lower binge eating prevalence associated with semaglutide and fewer binge episodes with liraglutide, effects of these medications on disordered eating are extremely limited overall and non-existent in children.^15,54,55^ There is similarly little empirical evidence on the effects of GLP-1 use on restrictive eating behaviors; however, it is also possible that medication-induced changes in these same appetitive processes could support increased dietary restriction.^56^ Furthermore, in line with theoretical models drawn from the eating disorders literature, rapid and large changes in body weight/size accompanied by external validation from peers, providers and/or family members may heighten appearance-related concerns and fear of weight regain, thus reinforcing potential restrictive eating behaviors.^57^

Based on these potential impacts, evidence to clarify the relationship between GLP-1 use and disordered eating behaviors or eating disorders is critically needed and widely sought. For example, in a nationwide survey, U.S. adolescents and young adults shared concerns that GLP-1 use could contribute to eating disorder risk, as well as their desire for more safety information.^58^ Similarly, pediatric primary care clinicians were less willing to prescribe anti-obesity medications such as GLP-1 to youth exhibiting signs and symptoms concerning for disordered eating.^59^ Finally, in a qualitative study of adolescents and young adults using or planning to use anti-obesity medications including GLP-1, as well as their caregivers, experiences related to disordered eating were variable, with one young adult sharing that she had become ‘addicted to weight loss’ after starting the medication. However, others shared positive experiences, noting medication use had resolved pre-existing binge eating and restriction behaviors, and had improved their relationships with food.^60^ Ultimately, the current evidence related to GLP-1 use and disordered eating behaviors or eating disorders in youth remains extremely limited and heterogeneous.

*Table 2* summarizes the medication regimens of participants in the included studies of youth with type 2 diabetes who responded to disordered eating questionnaires.^19–24,26^ Due in part to the more recent U.S. Food and Drug Administration approval of GLP-1 for pediatric use (liraglutide for type 2 diabetes in 2019), none explicitly commented on the use of GLP-1 by participants, and insulin use was variable or not specified. While some studies noted that insulin omission may be used to promote unhealthy weight loss, others did not specifically discuss the potential for medications to affect appetite, dietary intake, disordered eating or weight.^19–24,26^

**Table 2:** Medication regimens of participants in included studies evaluating disordered eating in youth with type 2 diabetes^19–24,26^

First author (year)	Population	Disordered eating measure	Medication regimen(s)	Discussion of medication(s) in relation to appetite, dietary intake, disordered eating or weight?
Lawrence (2008)^26^	SEARCH for Diabetes in Youth Study: youth and young adults with type 1 and type 2 diabetes; minority (519 [15.5%]) with type 2 diabetes	YRBSS, modified	38% of females and 34% of males used insulin	Insulin omission as a strategy to promote unhealthy weight loss Using diet aids more common among youth with type 2 diabetes than type 1 diabetes
TODAY Study Group (2011)^23^	TODAY study: youth with recently diagnosed type 2 diabetes	Y-EDEQ	Metformin only	No
Weinstock (2015)^22^	TODAY study: youth with recently diagnosed type 2 diabetes	Y-EDEQ	Distribution of metformin alone, metformin and rosiglitazone and/or insulin not specified	No
Nip (2019)^19^	SEARCH for Diabetes in Youth Study: youth and young adults with type 1 and type 2 diabetes; minority (149 [6.5%]) with type 2 diabetes	DEPS-R	All using insulin, due to the DEPS-R question about insulin omission	Insulin omission as a strategy to promote unhealthy weight loss
Eitel (2024)^20^	SEARCH for Diabetes in Youth Study: youth and young adults with type 1 and type 2 diabetes; minority (353 [22%]) with type 2 diabetes	DEPS-R	Not noted	No
TODAY Study Group (2022)^24^	TODAY study: youth with recently diagnosed type 2 diabetes	EDDS	Distribution of metformin alone, metformin and rosiglitazone and/or insulin not specified	Noted that insulin omission as a strategy to promote unhealthy weight loss was not assessed
Julceus (2025)^21^	SEARCH for Diabetes in Youth Study: youth and young adults with type 1 and type 2 diabetes; minority (395 [14.8%]) with type 2 diabetes	DEPS-R	Not noted	Insulin or oral hypoglycemic drug omission as a strategy to promote unhealthy weight loss Glucagon-like peptide-1 receptor agonists not mentioned

*Note: All studies except for Eitel et al. and Julceus et al. included data collected prior to the first U.S. Food and Drug Administration approval of glucagon-like peptide-1 receptor agonists for youth (liraglutide in 2019).*

*DEPS-R = Diabetes Eating Problem Survey-Revised; EDDS = Eating Disorder Diagnostic Scale; TODAY = Treatment Options for type 2 Diabetes in Adolescents and Youth; Y-EDEQ = Youth-Eating Disorder Examination Questionnaire; YRBSS = Youth Risk Behavior Surveillance System.*

## Clinical strategies to minimize harms related to disordered eating behaviors and eating disorders in youth with type 2 diabetes

Given the current very sparse evidence base, it is conservative but reasonable to assume that youth seeking and using GLP-1 for type 2 diabetes management are at increased risk for disordered eating due to the guideline-recommended emphasis on weight management as a key aspect of type 2 diabetes treatment.^61^ Until a validated screener for this population is available, providers should remain cognizant of signs or patterns of disordered eating behaviors that may emerge. It is important to note that such patterns may closely resemble the normative response to treatment but may be greater in pace or magnitude, such as losing weight more quickly than recommended, the inability to stop losing weight once a target weight is reached, engaging in dieting that becomes progressively more restrictive over time, food avoidance without medical, ethical or cultural reason, regularly skipping meals, becoming preoccupied with eating and sudden and extreme changes in exercise behavior.^62^ Other symptoms and behaviors that should raise concern for eating disorders include feeling a loss of control over what or how much is eaten, vomiting for shape or weight reasons and laxative or diuretic use. Providers are encouraged to monitor disordered eating symptoms before GLP-1 initiation, regularly throughout treatment and after discontinuation using validated screening tools such as the EDDS or mSCOFF, while recognizing that these tools may not detect the full spectrum of risk for disordered eating among youth with type 2 diabetes, including those using GLP-1 medications, and may require supplemental assessment. New onset or worsening symptoms should signal more intensive monitoring and/or referral to a specialty provider for comprehensive evaluation.

One important way to support youth at risk for disordered eating is to minimize exposure to medical weight stigma, which has been shown to be associated with negative health outcomes across several domains, including elevated risk for eating disorder symptoms.^63,64^ Even well-intentioned providers may perpetuate weight stigma when caring for patients with overweight or obesity, especially in the context of providing weight management recommendations. Clinicians should practice awareness of their own biases around weight and the ways in which weight stigma manifests to provide high-quality assessment and care to individuals with higher weight.^62^ For all patients, personalized and collaborative goal-setting on health- and weight-related goals can be protective against the risk of eating disorders. We advise patient education about the harms of misusing GLP-1 (e.g. on-going GLP-1 use despite severe gastrointestinal side effects or appetite suppression that leads to prolonged fasting) and omitting insulin (i.e. worsening glycemic control, diabetic ketoacidosis). Lastly, consistent with clinical practice guidelines, clinicians should collaborate with dietitians and therapists in caring for youth with type 2 diabetes.^7^ Similarly, clinicians specializing in eating disorder treatment may benefit from support from the clinical diabetes team to understand the potential weight- and appetite-altering effects of medications, as well as dietary guidelines for type 2 diabetes management.

## Conclusion

Although further studies are needed, current evidence suggests that disordered eating behaviors are common in adolescents with type 2 diabetes, a risk that may be exacerbated by a strict focus on weight reduction as a treatment goal. More data are needed to understand the risks and benefits posed by the use of GLP-1 in this population, particularly related to disordered eating risk. Given the lack of validated screening tools for youth with type 2 diabetes and the serious consequences associated with unrecognized disordered eating, clinicians should be aware of the higher risk of disordered eating behaviors and eating disorders in this population, as well as the need to monitor for changes in behaviors during the use of GLP-1.
